# Hydrothermal Liquefaction of Digestate and Copyrolysis of Hydrochar–Straw Blends: An Integrated Approach for Bio-crude and Biochar Production

**DOI:** 10.1021/acs.energyfuels.6c01322

**Published:** 2026-07-17

**Authors:** Corinna Maria Grottola, Giusy Marotta, Davide Amato, Francesca Di Lauro, Marco Balsamo, Fabio Montagnaro, Roberto Solimene, Raffaele Ragucci, Paola Giudicianni

**Affiliations:** † Istituto di Scienze e Tecnologie per l’Energia e la Mobilità Sostenibili (CNR-STEMS), Naples 80125, Italy; ‡ Dipartimento di Scienze Chimiche, 98793 Università degli Studi di Napoli Federico II, Naples 80126, Italy; § Dipartimento di Ingegneria Chimica, dei Materiali e della Produzione Industriale, Università degli Studi di Napoli Federico II, Naples 80126, Italy

## Abstract

Anaerobic digestion converts wet biogenic wastes into biogas and a byproduct, the digestate, which is a wet material rich in carbon and nutrients widely used in agriculture. However, the large amount produced determines some limitations in this exploitation, thus different thermochemical processes are under investigation. The hydrothermal liquefaction (HTL) process is considered a viable thermochemical route to convert high-water-content biomasses into a liquid energy vector, the bio-crude. However, considerable uncertainties remain regarding the use of the HTL solid residue, the hydrochar (HC), which is not regulated as soil amendment. Thus, its transformation into biochar, already recognized by law as soil improver, could be a promising valorization route. In this context, copyrolysis of HC with abundant agricultural residues may represent an effective strategy to further improve resources’ valorization. This study proposes an integrated approach that combines the conversion of digestate through HTL into bio-crude and copyrolysis of hydrochar and straw (S), an abundant agricultural waste to produce biochar. HTL of digestate has been performed in a 500 mL batch autoclave reactor and optimized, based on the biocrude yield and properties, investigating different temperatures (300 and 350 °C) and isothermal reaction times (0–30 min). HC produced under the optimal operating conditions for bio-crude production and S have been pyrolyzed individually and in blends with varying compositions (10–50 wt % S) under slow pyrolysis conditions at a final temperature of 450 °C. Pyrolysis products’ yields and properties of biochars have been investigated to assess the compliance of biochar with EU Fertilizing Products Regulation (EU 2019/1009) and to identify possible nonlinear effects arising from the combination of the two feedstocks. Results show high biochar yields in the range of 42.8–62.9 wt % and high H/C ratios in the range of 0.68 to 0.61 which comply with the threshold limits imposed by the EU regulations.

## Graphical abstract

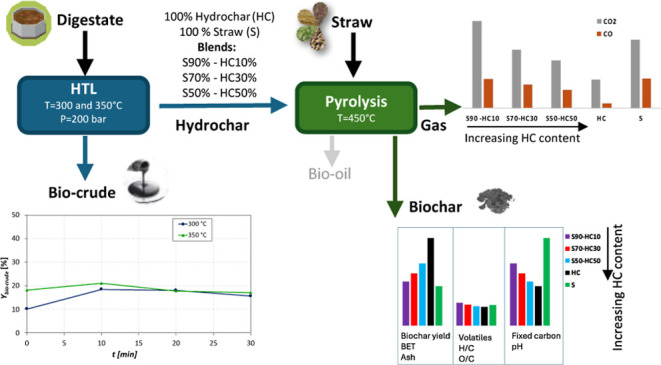

## 1 Introduction

The current world energy system still depends mainly on fossil sources, and according to projections by the International Energy Agency (IEA), their consumption is expected to reach its peak before 2030 (IEA 2025). As a consequence, a significant increase in greenhouse gas (GHG) emissions is still expected in the next few years, substantially contributing to climate change. In this context, the revised Renewable Energy Directive (RED III) sets ambitious decarbonization targets, reducing the GHG intensity from the transport sector by 14.5% or increasing the use of renewable energy sources in final energy consumption.^1^ In the same line, developing energy-efficient, zero-waste biorefineries can reduce the production costs of advanced biofuels for transportation while enabling the coproduction of high-value biobased products, supporting both the energy demand and climate target.^2^ Focusing on the second biomass generation type, including residues from organic and food waste and lignocellulosic biomass (agricultural residue), for advanced biofuel and biobased material production, the sustainable valorization routes are still limited due to lower product yields and the valorization pathways for the side stream.^3,4^

As for organic waste, anaerobic digestion (AD) represents the most established technology to produce biogas suitable for electricity generation.^5,6^ However, one of the main challenges of this process is the large amount of digestate produced, which still contains around 50% of the original organic matter,^7,8^ and that should be appropriately valued, since its transportation becomes economically unfavorable for distances longer than 10 km.^9^ Depending on the feedstock and AD conditions, the digestate can be used in agriculture or further processed for nutrient recovery or energy production.^10,11^ Even so, due to the high moisture content of digestate, typically in the range of 75–95 wt %, an energy-intensive drying step is required before any thermochemical process, resulting in decreased energy efficiency. In this context, the hydrothermal liquefaction (HTL) process offers a way to overcome this limitation. HTL is a promising process that enables the conversion of organic substrates into bio-crude in liquid water under subcritical conditions, at temperatures and pressures of 250–374 °C and 40–250 bar, respectively.^12–14^ Under subcritical conditions, water favors the solubilization of the organic fraction of biomass and allows the breakdown of the macro-components present therein.^15–17^ In this way, it would be possible to convert a fraction of the carbon in the digestate into bio-crude that, appropriately upgraded, can produce liquid fuels for the transportation sector. This bio-crude typically contains ∼10–15 wt % heteroatoms (mainly O and N) with an ∼30–40 MJ/kg higher heating value (HHV). HTL bio-crude is receiving increasing interest as a promising biobased carbon vector that by further upgrading processes, such as hydro-deoxygenation (HDO) and hydro-denitrogenation, can produce Sustainable Aviation Fuels (SAFs). In this way, heteroatoms can be removed and/or specific chemical transformations can be promoted to obtain the desired classes of aromatics, cycloalkanes, iso-alkanes, and *n*-alkanes.^18^ Preliminary studies have already demonstrated the possibility of achieving good energy recovery from the HTL of digestate.^7,8,19–21^ It has been reported in the literature that bio-crude is generally rich in phenolic compounds, fatty acids, and N-heterocyclic species.^22^ However, HTL also generates coproducts, including a solid residue known as hydrochar, a low-grade gas phase, and an aqueous phase, which must be appropriately managed and/or valorized.^23^ In particular, hydrochar represents a coproduct with yields typically ranging from 20 to 45 wt %.^24^ Hydrochar is rich in carbon and ash (around 65–75 wt %), in nutrients, particularly some cases in phosphorus,^24^ but it is not under regulation for its use as a soil amendment, and further refinements are necessary, particularly in the ratio of the mineral fraction.^25,26^ To date, most of the studies focused either on integrated conversion routes involving digestate via hydrothermal carbonization (HTC) and HTC-derived hydrochar valorization through pyrolysis or on HTL of digestate followed by physicochemical characterization of the resulting hydrochar, without further upgrading via thermochemical processes. Research specifically focused on the pyrolysis of hydrochar derived from HTL of digestate remains scarce.

Pyrolysis is a well-consolidated and promising process due to its ability to valorize dry biomass and agricultural residues, abundant and inexpensive sources. By tuning the pyrolysis operating conditions properly (temperature, heating rate, and residence time), the biomass is converted into target pyrolysis products such as the liquid phase (bio-oil), the gaseous phase, and the solid, known as biochar. Biochar is a nutrient-carbon-rich and porous material that is receiving attention, and it is expected to play a growing role in the coming years, driven by the increasing adoption in agriculture for soil improvement, in soil bioremediation, and carbon sequestration.^27,28^ Recently, the use of biochar has also spread to other sectors due to its chemical-physical properties, such as its surface chemistry, which makes it suitable for the adsorption of pollutants,^29,30^ or as a filler in composite materials^31^ and in medicine application with antimicrobial activity.^32^ Feedstock composition and pyrolysis temperature are the main conditions that affect the biochar yield and properties.^33^

Up to now, the design and optimization of biorefineries have been based on the integration of pyrolysis with other processes, which have focused on one main product, bio-oil or biochar from one specific feedstock.^34^ Recent studies show the copyrolysis of lignocellulosic biomass with plastics or algae, to produce higher quality liquid fuels,^35,36^ with sludge to improve the energy density and manage ashes,^37–39^ and with hydrochar from HTC to improve the biochar quality in terms of nutrients available for plants and surface area.^40,41^ The behavior and valorization potential of HTL-derived hydrochar under pyrolytic conditions are still insufficiently understood, both hydrochar alone or in copyrolysis with lignocellulosic biomass.

Although valorization of digestate by HTL and of straw by pyrolysis is frequently reported in the literature, the specific combination of HTL and pyrolysis of digestate has been explored to a much lesser extent. Following the biorefinery concept, it is important to integrate biomass conversion processes and equipment to produce fuels, power, and/or value-added chemicals from wastes. In this context, this study is focused on the scenario of exploitation of digestate through the HTL process for bio-crude production and copyrolysis of hydrochar and straw, lignocellulosic biomass representative of agricultural waste, to have an optimization of the overall process efficiency in terms of the digestate valorization (Figure 1). For this purpose, optimal conditions of HTL are identified based on the bio-crude yields and composition. Hydrochar obtained under the optimal HTL condition has been blended in different ratios with straw to have a guideline to identify a proper blend ratio and produce biochar. Biochar obtained from the different blends was characterized to determine whether it can be used as a soil conditioner and whether it complies with the values specified in the threshold limits reported in Regulation (EU) 2019/1009 for fertilizers.

**Figure 1:**
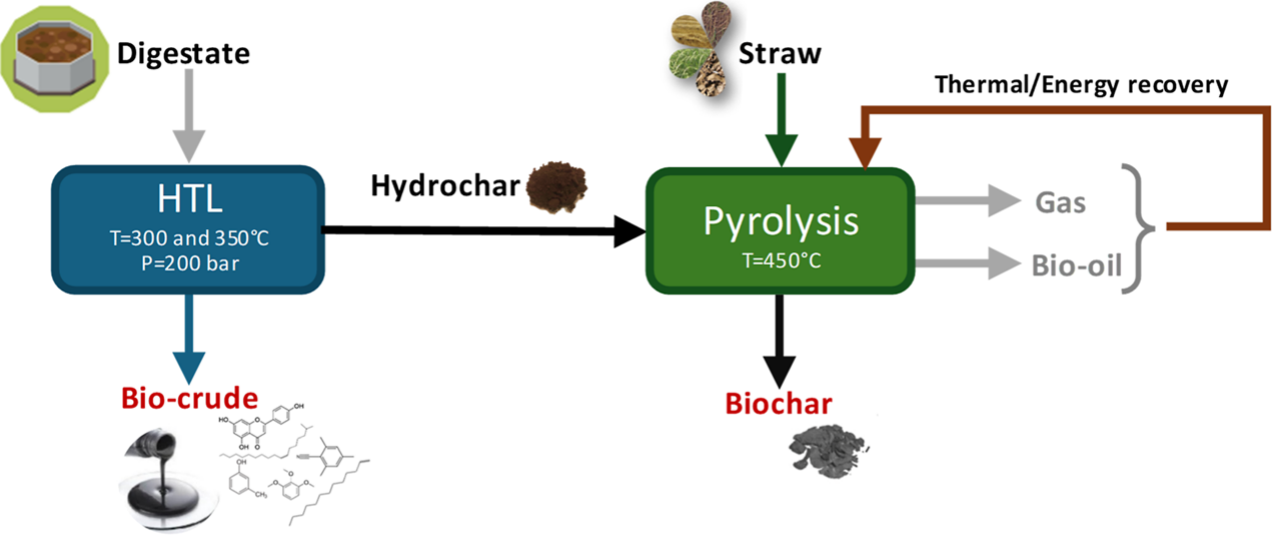
Conceptualization scheme of the integrated approach for bio-crude and biochar production.

## 2 Materials and Methods

### 2.1 Feedstock Characterization

In this section, the characterization of the feedstock used for HTL and pyrolysis experiments are reported. The digestate (D) taken as a reference substrate for HTL tests was obtained from anaerobic digestion of bovine manure produced in an agricultural context of southern Italy. Its moisture content, as received, was equal to 83.2 ± 0.05 wt %, in line with the value of typical biomass-derived digestate. For the pyrolysis tests, wheat straw (S) from an agricultural site in the province of Naples (Acerra), in the Campania region, was used as feedstock. For both feedstocks, D and S, the same mechanical pretreatments, grinding and sieving, were applied to obtain a powder with a particle size below 400 μm for the digestate and in the 400–600 μm range for the straw. Before HTL and pyrolysis tests, the feedstocks were dried in an oven at 105 °C for 24 h to remove the moisture content therein.

Both feedstocks were preliminarily characterized by (i) proximate analysis using a TGA701 LECO thermobalance (UNI 9903/ASTM D5142, and ASTM E870 standard for S sample); (ii) ultimate analysis by a LECO CHN628 analyzer (ASTM D5373) for C, H, and N determination and by a LECO SC-144DR analyzer (UNI 7584) for S determination. Oxygen content was calculated by difference, based on the measured C, H, N, S, and ash content, all expressed on a dry basis (db); and (iii) inorganic compound determination by inductively coupled plasma–mass spectrometry (ICP–MS) using an Agilent Technologies 7500ce instrument, after microwave-assisted acid digestion of the samples, following US EPA Methods 3052. Three repetitions were carried out, and the mean value is listed with the standard deviation in Table 1 as weight percentage on a dry basis (db) or dry and ash-free basis (dafb).

**Table 1:** Main Properties of Feedstock Used in HTL Experiments^a^

	[wt % db]			[wt % dafb]				
	Volatile	Ash	Fixed carbon	C	H	N	S	O^b^
D	50.1 ± 1.5	35.0 ± 2.6	14.9 ± 1.1	56.4 ± 0.3	7.0 ± 0.05	3.7 ± 0.1	0.9 ± 0.3	32.0 ± 2.3
S	70.7 ± 0.2	11.8 ± 0.2	19.5 ± 0.5	47.7 ± 0.5	5.8 ± 0.1	0.06 ± 0.1	1.1 ± 0.4	46 ± 0.6

Inorganic elements content [mg/kg, db]		
	Digestate	Straw
Na	3288 ± 3	1001 ± 1
Mg	6770 ± 2	522 ± 0
Al	7942 ± 4	448 ± 6
P	7342 ± 6	1525 ± 1
K	18906 ± 3	10025 ± 3
Ca	22346 ± 2	972 ± 3
Cr	14 ± 7	1.1 ± 5
Mn	219 ± 2	32 ± 0
Fe	3944 ± 3	245 ± 5
Ni	6 ± 5	0.7 ± 7
Cu	20 ± 5	15 ± 7
Zn	116 ± 6	45 ± 7
Pb	4.6 ± 2	0.7 ± 4
Cd	2.9 ± 0.5	0.6 ± 0.01

a In the table, the acronyms “db” and “dafb” refers to data reported on a dry and on a dry and ash-free basis, respectively.

b % O calculated as 100 – (C + % H + % N + % S).

The proximate analysis shows a significant ash content, reflecting the presence of inorganic nutrients typically concentrated during anaerobic digestion. While some of these minerals, namely, K and P, are present in high amounts thus contributing to the fertilizing value of digestate, high levels of toxic elements, such as Cd exceeding the EU regulation limit of 1.5 mg/kg (db), may prevent its application to agricultural lands. The ultimate analysis indicates a moderate carbon content together with the presence of N and S, confirming the residual organic matter and nutrient content of the digestate. However, considering the relative content of N (2.4 wt %), K (∼1.9 wt %), and P (∼0.7 wt %), when applied directly to agricultural land, the nutrient balance may not always correspond to crop demands, potentially requiring integration with other fertilizers. On the other hand, the presence of a moderate amount of carbon (56.4 wt %) confirms that digestate represents an attractive feedstock for HTL. In addition, the inorganic fraction may promote catalytic effects during HTL reactions, potentially enhancing the conversion of organic matter into bio-crude. Therefore, despite the limitations associated with its direct land application, digestate represents a promising feedstock for HTL-based valorization pathways.

### 2.2 HTL Experimental Setup and Products’ Separation Protocol

For the experimental campaign, HTL tests were performed at temperatures of 300 and 350 °C and times ranging from 0 to 30 min to investigate the effect of temperature and reaction time on the distribution of HTL products.

A detailed description of the HTL experimental apparatus used in this work is reported in Di Lauro et al.^42^ Briefly (Figure 2), it is a 500 mL batch reactor (Parr Instruments, series PA 4575A) equipped with a digital pressure transducer coupled with a needle valve for pressure measurement and control, two aluminum semicylindrical heating blocks coupled with thermocouples and a PID system for temperature setting, measurement, and heating rate control systems. To reduce the heating stage, the system was also equipped with a 1250 W band heater (Watlow Series MI band), coupled with a cylindrical steel block located at the bottom of the vessel and with a layer of rock wool at the top of the reactor. In this configuration, the heating rate was about 8 °C/min to reach a temperature of 350 °C.

**Figure 2:**
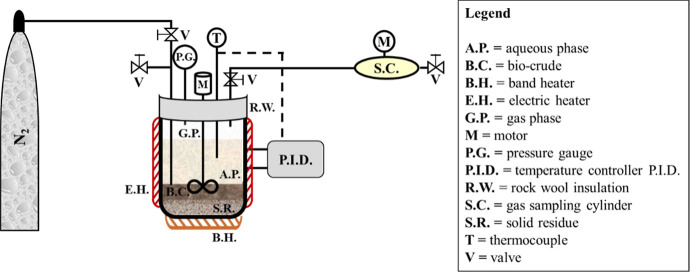
Layout of the 500 mL lab-scale batch autoclave reactor.

For all the HTL experimental tests, 30 g of dry digestate is mixed with 270 g of distilled water to obtain a slurry with a 10 wt % of solid content. The slurry was loaded into the reactor, which is sealed and subsequently insulated with rock wool. The two-aluminum semicylindrical heating blocks are closed around the reactor walls, and the head space of the reactor is purged with N_2_. Then, the system is pressurized to obtain a pressure of approximately 200 bar at the set point temperature chosen for the reaction. Finally, the stirrer inside the reactor is activated at a speed of 600 rpm and the heating system turned on to begin the HTL test.

Once the heating ramp has been completed and the preset reaction time has elapsed, a fast cooling of the reactor is carried out, to return at ambient temperature, and the overpressure in the reactor, compared to the initial one, allows us to quantify the mass of gas produced during the process. Finally, the gas phase was vented to the atmosphere to restore ambient pressure and allow reactor discharge.

The experimental protocol for the recovery of HTL products was originally defined in Di Lauro et al.^25^ After the HTL test, the liquid and solid phases were recovered from the vessel using about 30 g of dichloromethane (DCM). The resulting slurry was filtered on a Büchner funnel under vacuum to obtain a solid phase and a water/bio-crude mixture. The solid phase was subjected to Soxhlet extraction to recover the bio-crude from the solid pores using DCM as the extracting solvent. Instead, the water/bio-crude mixture was separated by centrifugation and stratification between the aqueous and the oily phase.

At the end of these operations, the hydrochar was oven-dried at 105 °C for 24 h while the soluble fraction in DCM obtained during Soxhlet extraction was joined with the bio-crude resulting from the liquid–liquid separation. Finally, the solution of bio-crude and DCM was distilled under vacuum to remove the DCM from the bio-crude.

After the separation processes, the bio-crude (BC), hydrochar (HC), gaseous (G), and aqueous phase (AP) yields (*Y*) were calculated according to eqs 1–4, respectively.

1 $$Y_{gas}^{db}=\frac{m_{gas}}{m_{biomass}^{db}}\times 100\;\text{or}\;Y_{gas}^{dafb}=\frac{m_{gas}}{m_{biomass}^{dafb}}\times 100$$

2 $$Y_{hydrochar}^{db}=\frac{m_{hydrochar}^{db}}{m_{biomass}^{db}}\times 100$$

3 $$Y_{bio‐crude}^{db}=\frac{m_{bio‐crude}}{m_{biomass}^{db}}\times 100\;\text{or}\;Y_{bio‐crude}^{dafb}=\frac{m_{bio‐crude}}{m_{biomass}^{dafb}}\cdot 100$$

4 $$Y_{water-soluble\;compounds}^{db}=100-\left(Y_{gas}^{db}+Y_{hydrochar}^{db}+Y_{bio‐crude}^{db}\right)$$

where *m*_gas_, *m*_solid residue_, *m*_bio‑crude_, and *m*_biomass_ represent the mass of gas, hydrochar, bio-crude, and starting biomass, respectively. The superscript “db” refers to a dry basis, while “dafb” refers to a dry and ash-free basis. It is assumed that the gas phase is made of only CO_2_, this being the major component of the mixture of produced gases, generally greater than 85%.^25,43^

### 2.3 Pyrolysis Experimental Setup

Pyrolysis tests were conducted by using an experimental setup consisting of a pyrolysis reactor, a condensation system for the collection of liquids, and an online gas analyzer.

The bench-scale reactor consists of a jacketed prismatic reaction chamber (*L* = 0.24 m, *W* = 0.04 m, *H* = 0.052 m) described in detail in previous works.^44^ About 6 g of feedstock is loaded in mm thick layers on 4 trays allocated uniformly along the rectangular cross section of the inner reaction chamber. Nitrogen flows into the jacket, heated at 5 °C/min, at a constant gas flow rate. The temperature is monitored using N-type thermocouples in three points along the longitudinal and cross sections of the chamber. The vapors, carried by the nitrogen flow, exit the reactor and pass through a cooling stage consisting of a jacketed coil cooled with water at 5 °C, where the condensable fraction is collected in a flask placed at the end of the condensation system. The noncondensable gases are analyzed online through a micro gas chromatography analyzer (Agilent 990 Micro GC) at a rate of one analysis every 125 s (corresponding to a temperature increase of about 10 °C).

The gas analysis system was calibrated by using specific gas mixtures containing known concentrations of H_2_, O_2_, N_2_, CO, CH_4_, CO_2_, and C_2_. The value of the N_2_ flow was used to convert the volumetric composition of the gas phase into the volumetric release rate curves of the gas species. By integration of the gas release rate, it was possible to calculate the produced gas volume (and mass) for each gas species.

The biochar yield was measured directly by weighing the remaining solid residues at the end of each run. Since the condensation system does not ensure reproducible liquid recovery, the liquid yield was calculated by difference to close the overall mass balance. The liquids are not part of this work. The reported yields represent the average values of at least two tests.

All the feedstocks were pyrolyzed at atmospheric pressure with a constant flow rate of nitrogen (3 Nl/min) and heated up to the final temperature of *T* = 450 °C at a heating rate of 5 °C/min. The biochar production temperature was selected to combine high carbon yield and long-term carbon sequestration in the biochar. Biochars with H/C lower than 0.7, typically obtained at high pyrolysis temperature,^33^ are generally considered stable over centennial time scales and associated with high carbon permanence.^45^ Even though increasing temperature enhances aromaticity and long-term stability, it reduces biochar yield due to greater devolatilization and carbon conversion into liquids and gases.^46^ The selected biochar production temperature represents a trade-off temperature balancing biochar stability with high carbon retention in the solid phase.

In this work, the pyrolysis products of each experiment in the case of single feedstocks will be referred to by the name of the feedstock: wheat straw (S) and hydrochar (HC), while for the blends, the feedstock names together with their weight ratios are used. The three S-HC blends investigated have S/HC ratios of 90%–10%, 70%–30%, and 50%–50%, respectively. The corresponding biochar (B) will be identified using the notation B-feedstock-ratio.

### 2.4 HTL and Pyrolysis Products’ Characterization

#### 2.4.1 Bio-crude

Bio-crudes obtained after the HTL test were characterized by an ultimate analysis analyzer and evaluation of HHV according to the standard procedure described in Section 2.1. Given the elemental composition and calorific value of the bio-crude, it was possible to evaluate the associated energy recovery:

5 $$ER=\frac{{HHV}_{bio‐crude}}{{HHV}_{biomass}}Y_{bio‐crude}^{db}$$

The chemical analysis of bio-crude was performed by means of gas chromatography (GC) coupled with mass spectrometry (MS), following a methodology modified from ref^47^. The GC (Agilent 7980A) was equipped with a DB 624 capillary column, which works with helium as a gas carrier, and was coupled with a 5975C VL MSD mass spectrometer. The bio-crude samples were highly diluted in acetone (∼10 wt % bio-crude in acetone from VWR chemicals, purity >99%) and the samples were filtered with 0.2 μm PTFE syringe filters before the injection in the GC–MS. The injection occurs through an autosampler: 1 μL of the sample was injected for each analysis, whose run time was about 118 min. The column was heated according to the following temperature program: 4 min at 45 °C, heating up to 235 °C with a heating rate of 3 °C/min and 50 min at 235 °C. The sample was injected in “split mode” and carried by a flux of helium of 1.8 mL/min. The detector was set to scan a range of m/Z between 35 and 350. Bio-crude volatile compounds’ identification was carried out by matching the obtained spectra with the National Institute of Standards and Technology (NIST) library. The results were expressed in terms of area percentage of the identified compounds with respect to the total area of the revealed compounds. The compounds with a quality match lower than 70 are reported as “unknown” in the characterization, but they were still included in the total area calculation. The detailed GC–MS characterization is reported in the Supporting Information.

Finally, the determination of inorganic elements was performed by ICP–MS following the same procedure as that adopted in Section 2.1.

#### 2.4.2 Hydrothermal Gas

For specific HTL tests, the composition of the gas phase was determined through gas chromatography. After the HTL test, the gas was sampled by purging it from the reactor head to a system made in sequence, of a bubble column, a U-shaped adsorption column, and a 3 L gas sampling bag (Tedlar PLV Gas Sampling Bag, Thermogreen LB-2 Septa). The bubble column is filled with a 1 M acidic solution of H_2_SO_4_ to trap basic compounds like ammonia while the U-tube contains anhydron for the moisture adsorption. Finally, the gas samples were analyzed by a micro GC following the same procedure adopted in Section 2.3. The gas analysis allows to determine the concentration of light hydrocarbons (C_1_–C_6_) at concentrations up to the order of parts per million, in addition to CO, CO_2_, CH_4_, H_2_, and O_2_.

#### 2.4.3 Solid Products’ Characterization: Hydrochar and Biochar

All the solid products from HTL and pyrolysis were characterized in terms of proximate analysis, ultimate analysis, and determination of inorganic element content following the same procedure adopted for feedstocks described in Section 2.1. The pH of the biochar was determined by using a digital pH meter (827 pH LAB, Metrohm) in deionized water at a 1:20 weight-to-weight ratio, following the ASTM D4972-13 standard method.

Porosimetry analysis was performed using nitrogen adsorption at −196 °C with an Autosorb-1 apparatus (Quantachrome). The specific surface area was calculated using the Brunauer–Emmett–Teller (BET) method. Specifically, the specific surface area was determined using the standard 11-point BET method (Autosorb iQ, Quantachrome), applying the typical relative pressure range of *P*/*P*_0_ = 0.05–0.30. Before analysis, samples were degassed at 300 °C for 5 h under vacuum.

Infrared spectra of the samples were obtained using a PerkinElmer Frontier spectrophotometer with the attenuated total reflectance (ATR) technique. This method enabled the analysis of the samples in powder form and did not require mixing with KBr. The ATR spectra were recorded in the 450–4000 cm^–1^ range using a zinc selenide (ZnSe) crystal, with a resolution of 4 cm^–1^. A total of 32 scans were collected for each sample, and background noise was corrected.

Three repetitions were carried out for all of the characterization analyses, and the mean value was listed with the standard deviation. However, for the elemental analysis of the blends, nine repetitions were performed for each biochar sample because the very small amount of material required by the analytical technique (15 mg) determined a high standard deviation of the results.

## 3 Results and Discussion

### 3.1 HTL Products’ Yield and Properties

Figure 3 shows the yields on a dry basis of the HTL product as a function of reaction time from 0 to 30 min, at two different temperatures, namely 300 and 350 °C. It can be observed that for both temperatures a high amount of the bio-crude is produced during the heating stage, corresponding to a yield of 10% and 18% for process temperatures of 300 and 350 °C, respectively. These bio-crude yield values represent approximately 54% and 86% of the bio-crude obtained under the best operating conditions in terms of yield (300 °C-10 min and 350 °C-10 min). So, the difference in terms of bio-crude obtained at 300 and 350 °C is evident only during the first 10 min of isothermal stage. After this reaction time, maximum bio-crude obtainable from the organic fraction is achieved. For longer reaction times, the yields of bio-crude decrease, while those of water-soluble compounds increase, indicating for longer times a biomass conversion toward the coproducts of the process and toward the undesired water-soluble compounds. This trend is consistent with the high content of nitrogen and oxygen compounds characteristic of the starting biomass, which tend to solubilize in the aqueous phase during the process, already starting from temperatures of 250 °C.^7,20^

**Figure 3:**
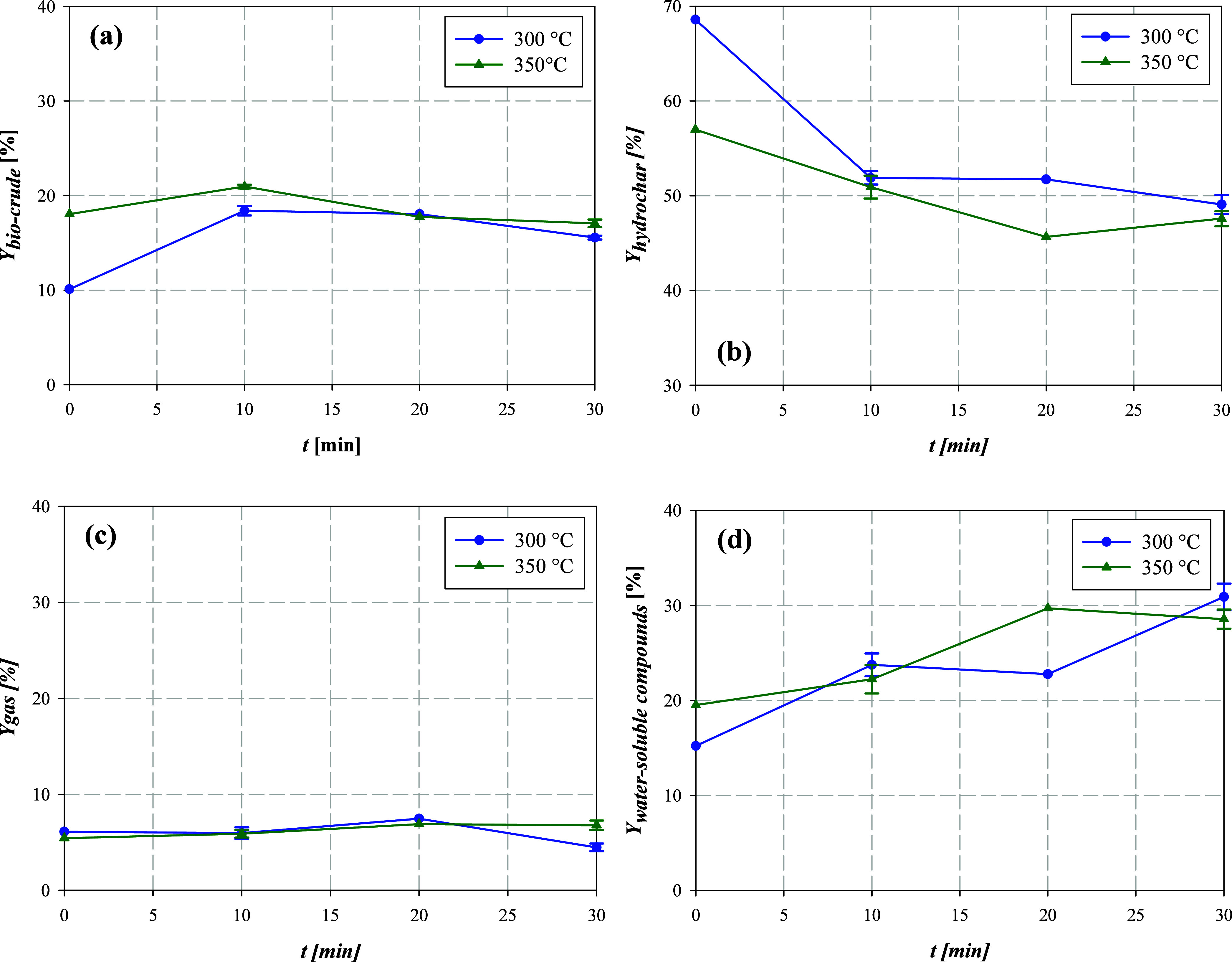
(a) Bio-crude, (b) hydrochar, (c) gas phase, and (d) water-soluble compounds yields after HTL tests as a function of time and for different temperatures. Yield values are given on a dry basis.

In particular, for the tests carried out at 350 °C, a bio-crude yield of about 21% (30% on a dry and ash-free basis) is reached at 10 min, then it slightly decreases to *t* = 30 min (*Y* ^db^_bio‑crude_ = 17%). A similar trend was obtained for tests carried out at 300 °C. The yield of bio-crude increases up to the isothermal reaction time of 10 min (*Y* ^db^_bio‑crude_ = 18.5%), remains more or less constant for *t* equal to 20 min (*Y* ^db^_bio‑crude_ = 18%), then it slightly decreases at 30 min where *Y* ^db^_bio‑crude_ is equal to 15.5%. Correspondingly, the yield of hydrochar from HTL of digestate, *Y* ^db^_hydrochar_, decreases with time during the isothermal stage from 69% to 49% at 300 °C up to 30 min, while from 57% to 45.5% at 350 °C, with an increase up to 47.5% at 30 min, indicating a possible reconversion of the products to form hydrochar for more severe reaction conditions. The high amount of solid residue is mainly due to the high percentage of ash in the starting digestate (cf. Table 1) as anaerobic digestion previously applied to the starting biomass reduced the organic content in the biomass derived-digestate and consequently increased the unconvertible inorganic fraction, which at the end of the HTL process is concentrated in the hydrochar, apart from water-soluble inorganic salts.^25^ Finally, the yield of the gas phase, *Y* ^db^_gas_, shows a slightly increasing trend from 5% to 7% at 350 °C during the isothermal stage.

Focusing on the quality of bio-crude, the bio-crude yield values and properties obtained under the different operating conditions adopted in this work are presented in Table 2. It can be observed that after the HTL process, there is a densification of C and H (as desired) in bio-crude with respect to the parent digestate, despite the starting biomass had already been partially valorized through anaerobic digestion. Moreover, the elemental analysis allowed us to calculate the values of the atomic ratios H/C and O/C, which are under the best operating conditions (350 °C and 10 min), equal to about 1.3 and 0.3. The liquefaction process, thus makes it possible to maintain the H/C and O/C ratio of the bio-crude comparable to the starting biomass (H/C = 1.5 O/C = 0.4) but strongly reduces the ash content, from the 35% on dry basis of the starting biomass to a value substantially negligible for the final bio-crude.^25^ This determines an increase of the higher heating value of approximately 2–2.4 times that of the starting biomass making bio-crude particularly attractive from an energetic point of view. Focusing instead on the energetic properties of the several bio-crudes investigated, the HHV values show small differences for all operating conditions (25–28 MJ/kg), thus (cf. Eq. 5) the difference in energy recovery (ER = 21–50%) is mainly determined by the greater value of bio-crude yield obtained at 350 °C and the reduced amount of oxygen for more severe process conditions. For longer reaction times and a higher temperature (i.e., 350 °C and 20–30 min), it is possible to observe that both the mass yield of the bio-crude and the C and H content are slightly reduced, resulting in a lower ER with respect to the best operating conditions. Moreover, a lower content of C and H results in an increase in the O content, leading to lower values of the H/C ratio and higher values of the O/C ratio, which make the bio-crude obtained at longer times more oxygenated and probably less stable than that determined under the best operating conditions. Focusing on the *S* content, in the present biomass case, though sulfur is not concentrated in the bio-crude, the level of its concentration is such that bio-crude cannot be used as it is in typical combustion applications, since it slightly exceeds the limits on S in fuels which are dictated by EU and international environmental regulations (% *S* < 0.1). Therefore, bio-crude upgrade processes would require a stage for the reduction of the *S* percentage under the normative limit.

**Table 2:** Main Properties for Bio-crudes Obtained after the HTL Process Conditions

*t*	*T*	*Y*_bio‑crude_^db^ (*Y*_bio‑crude_^dafb^)	C [%]	H [%]	N [%]	S [%]	O^b^ [%]	H/C [-]	O/C [-]	HHV [MJ/kg]	ER [%]
	Digestate	-	56.4	7.0	3.7	0.9	32.0	1.5	0.4	11.76	-
0	300 °C	10.1 (14.3)	56.3 ± 0.4	6.2 ± 0.2	3.0 ± 0.2	0.7 ± 0.3	33.8	1.3	0.4	24.68	21.2
10		18.4 ± 0.5^a^(26.1)	58.9 ± 0.5	6.1 ± 0.1	2.5 ± 0.1	0.8 ± 0.4	31.7	1.2	0.4	23.58	36.9
20		18.0 (25.6)	56.4 ± 0.5	5.6 ± 0.2	2.2 ± 0.1	0.8 ± 0.5	34.9	1.2	0.5	25.66	39.4
30		15.6 ± 0.2^a^(22.1)	57.8 ± 0.4	6.1 ± 0.2	2.4 ± 0.04	0.9 ± 0.5	32.8	1.3	0.4	26.56	35.1
0	350 °C	18.1 (25.6)	57.5 ± 0.6	5.9 ± 0.4	2.6 ± 0.2	0.7 ± 0.4	33.3	1.2	0.4	25.59	39.3
10		20.9 ± 0.2^a^(29.7)	64.0 ± 0.7	6.8 ± 0.5	2.4 ± 0.2	0.7 ± 0.4	26.1	1.3	0.3	27.85	49.5
20		17.7 (25.2)	57.4 ± 0.2	7.1 ± 0.2	2.6 ± 0.1	0.8 ± 0.3	32.1	1.5	0.4	26.63	40.2
30		17.1 ± 0.4^a^(24.2)	58.0 ± 0.4	6.6 ± 0.3	2.5 ± 0.1	0.9 ± 0.4	32.0	1.4	0.4	28.20	40.9

a As a mean of two HTL test replicates.

b % O calculated as 100 – (% C + % H + % N + % S).

The observed trend can be interpreted considering that the bio-crude represents an intermediate product of the HTL process. Therefore, while in a first phase, the increase in reaction times positively affects *Y*_bio‑crude_, composition, and energy recovery of bio-crude, on the contrary, it is reasonable to predict that even longer times lead to lower yields. As a matter of fact, given the series-parallel nature of the reactive network involved; longer reaction times increase the likelihood of condensation, cyclization, and repolymerization reactions between intermediate products, leading to biochar formation and a decrease in the quality of bio-crude.^48,49^

Figure 4 shows the main component families obtained from the GC–MS analysis performed on the digestate-derived bio-crude obtained under the best operating conditions in terms of yield and energetic properties achieved (the detailed list of all components identified by GC–MS is given in Table S1 of the Supporting Information). Similar GC–MS chromatograms are also obtained for the bio-crudes achieved under all operating conditions. The results were reported as normalized percentage area, but due to the limited GC–MS oven temperature (around 300 °C), only the light fraction of the bio-crude can be identified (usually between 10 and 20% wt.), while the high molecular weight compounds will not be detected.^50^ For manure digestate, the majority (∼48%) of the sum of the GC–MS peak areas for bio-crude oil produced by HTL was associated with compounds that were categorized as phenolic compounds whereas the rest were categorized in order of percentage as aldehydes/ketones (∼12%), N-heterocyclic compounds (∼11%), hydrocarbons (∼6%), and organic acids (∼4%). These results are consistent with the nature of starting biomass that is typically characterized by a more balanced distribution of lignin, carbohydrates, and crude lipids, which under HTL conditions of temperature produced a bio-crude that contained a mix of phenolic- and lipid-derived compounds. For example, it is likely that phenol compounds are derived from the carbohydrate and protein fractions, and similarly crude protein and the reactions between proteins and carbohydrates (Maillard reactions) present in the parent manure led to nitrogenous compounds, including succinimide, indole, and pyrazine.^22,51,52^ It is also important to emphasize the high presence of heteroatoms; the majority fraction of the bio-crude obtained from the starting biomass of this study represents a major problem, particularly in the low-boiling fraction traditionally used for the production of transport fuel.

**Figure 4:**
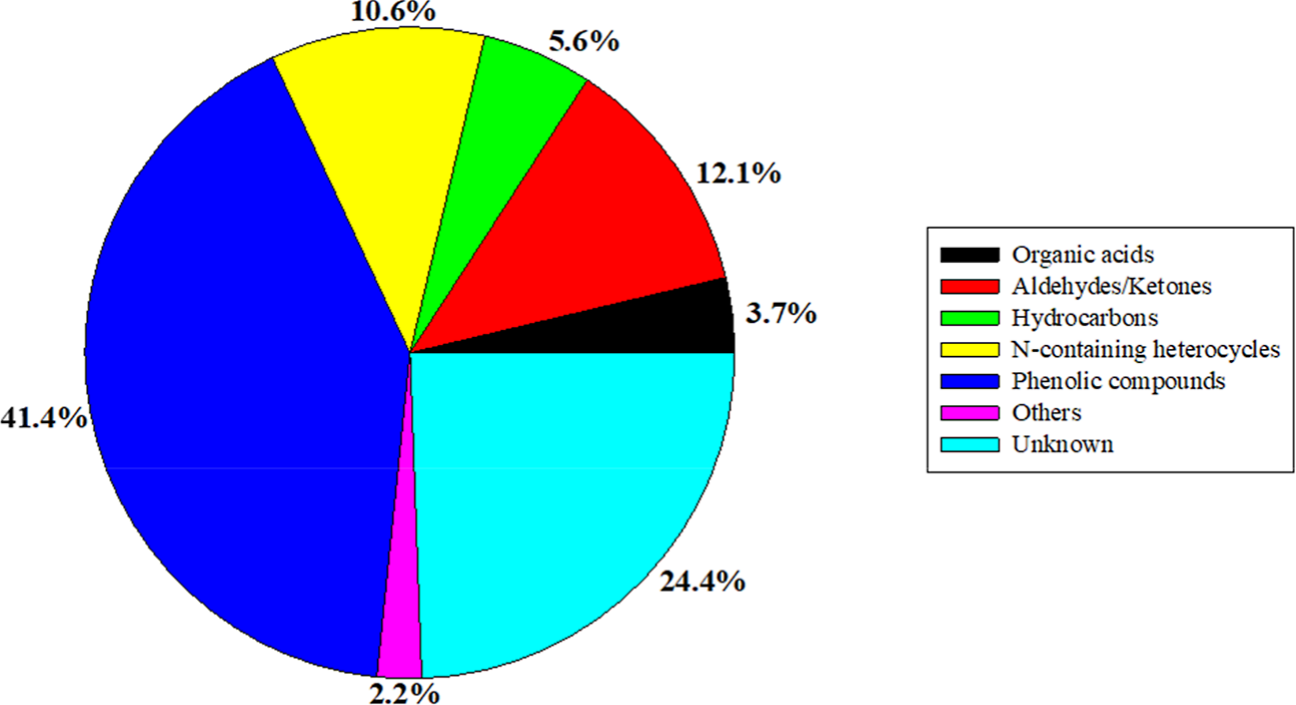
Distribution of the main organic compound classes of bio-crude obtained for a temperature of 350 °C and a reaction time of 10 min, identified by GC–MS analysis.

However, three of the four main organic families that make up the bio-crude analyzed (phenolic and aldehydes/ketones and hydrocarbons) are particularly attractive for SAF production, if suitably upgraded. For example, phenolic compounds represent a significant but problematic fraction of the bio-oil obtained from the HTL process, as the high oxygen content reduces the quality of the fuel, making it unstable and corrosive. These compounds can be converted into SAF through hydrodeoxygenation processes. This process, which is the most mature and widely used technology for “cleaning” the heavy fractions of bio-oil, uses high-pressure hydrogen and metal catalysts to remove oxygen from phenolic compounds in the form of water. After the removal of heteroatoms, the aromatic rings are often saturated to convert them into cycloalkanes, which give SAF optimal energy and density properties for aeronautical use.^18,53^ Similarly, oxygen-containing heterocyclic compounds, and in particular furans such as furfural and hydroxymethylfurfural (HMF), are considered ideal building blocks for SAF production and are often preferred over phenols due to their chemical structure, which makes the deoxygenation process easier. In fact, unlike phenolic compounds, which require hydrogenation of the rigid aromatic ring before the C–O bond can be broken, furans can undergo hydrogenation of the double bond with ring opening and final hydrogenation into alkanes, generally requiring less energy and less hydrogen than the saturation of stable aromatic rings. Instead, hydrocarbons can be easily upgraded through hydrocracking processes.^52,54,55^

To improve the efficiency of the HTL process, the coproducts of the process obtained under the best operating conditions in terms of yield, quality, and energy recovery of the HTL target product were characterized. Table 3 shows the yield and composition of the gas phase obtained at 350 °C and 10 min, while the full characterization of the corresponding hydrochar will be reported in the pyrolysis dedicated paragraph. As can be seen, the gaseous phase represents only a minor fraction of HTL products and consists almost exclusively of CO_2_, with minor percentages of CH_2_, H_2_, CO, and C_1_–C_4_. The low mass yield of the gas phase, together with its high CO_2_ content, makes this product unattractive. In an integrated HTL + pyrolysis system, it might be worth considering combining both gases generated by the processes to thermally support the integrated valorization system.

**Table 3:** Yield and Composition of the Gaseous Phase Obtained at 350 °C and 10 min

	Gaseous phase composition				
*Y*_gas_	[% vol. on a dry and N_2_-free basis]				
[% wt db]	CO_2_	H_2_	CH_4_	CO	C_1_–C_4_
5.9 ± 0.6	96.2 ± 0.4	0.8 ± 0.05	1.7 ± 0.3	0.7 ± 0.1	0.6 ± 0.02

### 3.2 Pyrolysis Products’ Yields and Properties

In this section, the pyrolysis experiments conducted on S-HC blends at different weight ratios are presented and discussed. The hydrochar used in the tests was obtained under the HTL best operating conditions for bio-crude production, namely, *T* = 350 °C and *t* = 10 min and was therefore selected for further valorization. Figure 5 reports the pyrolysis product yields obtained at 450 °C from S and HC and their blends. HC after pyrolysis exhibited the highest biochar yield, reaching 89 wt % db, due to the high ash content of the raw material, 55.53 wt % db (see Table 4). Only a limited formation of liquids and permanent gases was observed, achieving comparable values, consistent with the low volatile content of HC, indicating that a substantial fraction of the original organic matter had already been converted into bio-crude during the previous HTL treatment. HC pyrolysis results in high solid production due to both its high inorganic content and higher aromaticity compared to untreated biomasses, as evidenced by the lower H/C ratio of HC compared to S (Table 4). Potassium and sodium, which have well-known catalytic effects during pyrolysis,^46,56^ promote devolatilization of lighter volatiles and enhance cracking and reforming of vapors, producing lower liquid and gas.^57^ At the same time, previous findings show that the organic phase of hydrochar derived from digestates treated via hydrothermal liquefaction is composed of monocyclic, heterocyclic, and polycyclic aromatic structures, due to the dehydration, decarboxylation, and condensation reactions occurring during the hydrothermal process.^12^

**Figure 5:**
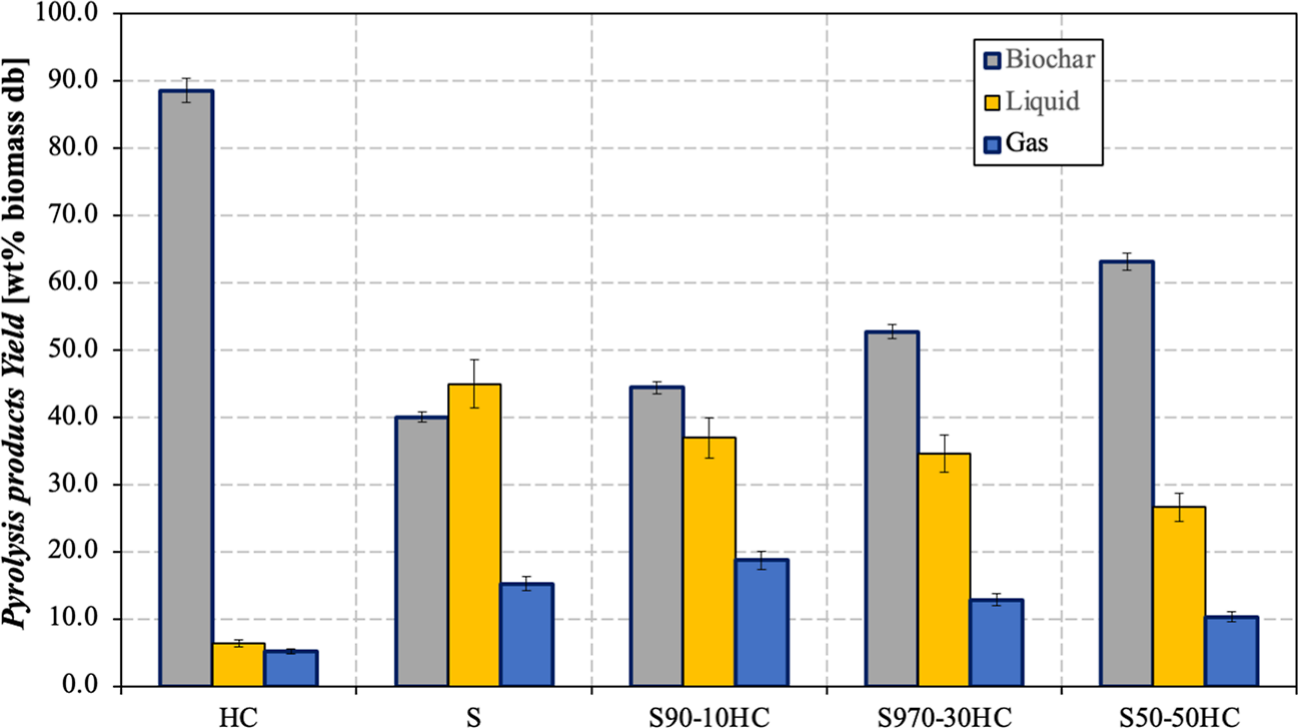
Yields of pyrolysis products at *T* = 450 °C, calculated on biomass db.

**Table 4:** Proximate and Ultimate Analysis, pH, and BET of S, HC, and all Biochars Produced from Pyrolysis Tests at 450 °C

	Volatile	Ash	Fixed carbon	C	H	N	O	S	H/C	O/C	pH	BET
	wt % db			wt % dafb								m2/g
HC	24.6 ± 0.5	55.5 ± 0.2	19.8 ± 1	62.3 ± 0.9	4.5 ± 0.2	3.7 ± 0.06	29.2 ± 1	1.15 ± 0.02	0.88	0.35	8.5 ± 0.05	21.84
S	70.7 ± 0.2	11.8 ± 0.2	19.5 ± 0.5	47.7 ± 0.5	5.8 ± 0.1	0.11 ± 0.01	46 ± 0.6	0.40 ± 0.07	1.48	0.73	-	-
B–HC	18.9 ± 0.3	61.7 ± 0.1	19.2 ± 0.7	76.37 ± 0.7	4.3 ± 0.04	6.5 ± 0.1	12.7 ± 0.9	1.2 ± 0.01	0.69	0.13	8.4 ± 0.05	64.58
B–S	20.8 ± 0.2	29.9 ± 0.7	49.1 ± 0.3	83. ± 0.5	3.7 ± 0.06	0.9 ± 0.03	11.9 ± 0.5	0.33 ± 0.03	0.54	0.11	10.9 ± 0.05	7.65
B–S90–HC10	23.1 ± 0.5	33.1 ± 1	43.6 ± 0.7	77 ± 2.06	3.92 ± 0.36	1.15 ± 0.1	17.9 ± 7.3	0.37 ± 0.01	0.61	0.17	10.7 ± 0.02	10.44
B–S70–HC30	2.0 ± 0.2	42.1 ± 0.1	36.8 ± 0.3	78.7 ± 0.4	3.6 ± 0.01	1.6 ± 0.05	16.0 ± 0.4	0.49 ± 0.01	0.56	0.15	9.6 ± 0.05	19.23
B–S50–HC50	19.6 ± 0.1	49.5 ± 0.2	30.7 ± 0.1	79.2 ± 1	3.9 ± 0.2	2.3 ± 0.07	14.4 ± 2	0.71 ± 0.0	0.59	0.14	9.1 ± 0.01	30.23

In contrast, S, despite having a fixed carbon content comparable with that of HC, produced a lower biochar yield of 40 wt % db, approximately half of that obtained from HC, due to the lower ash content in the raw material compared to HC. Accordingly, with the higher volatile content of the initial material, straw pyrolysis resulted in higher yields of liquid and gaseous products, amounting to 45 wt % db and 15 wt % db, respectively, with a liquid to gas ratio 3 times higher than that obtained from HC pyrolysis. As for the blends, copyrolysis of straw with hydrochar at different ratios resulted in intermediate product distributions. Biochar yields increase as the hydrochar fraction increases in the blends, ranging from 44 wt % for B-S90-HC10 to 63 wt % for B-S50-HC50, resulting in a decrease in the liquid yields. In contrast, gas yield has a nonmonotonic trend peaking at an S to HC ratio of 90:10. These trends suggest that some deviations from an additive dependence of product yields on blend composition may occur due to interactions between straw and hydrochar during copyrolysis. In particular, the high ash content of hydrochar may exert a catalytic influence on both primary devolatilization and secondary vapor-phase reactions that increase biochar yields, reduce condensable liquid yields, and shift part of the volatiles toward permanent gases. This behavior is observed for the S90–HC10 sample, which exhibits liquid and gas yields of 37 and 18.7 wt %, respectively. The liquid yield is lower and the gas yield slightly higher than the values predicted by an additive model (41 wt % for liquids and 14.2 wt % for gases), suggesting a deviation from ideal additive behavior. At higher concentrations of HC in the blend, the effect of inorganic materials achieves a plateau.

### 3.3 Biochar Properties

In Table 4, the main characteristics of all biochars produced are listed, including HC from HTL produced under the best conditions for bio-crude production. For the sake of clarity, in the discussion of the biochar properties, the characterization of S is also reported in Table 4. The HC before pyrolysis exhibits relatively low fixed carbon and C content (27.7 wt % on db) and high ash content, reflecting the effect of the previous thermal treatment during the HTL process. These results confirm literature results showing that HTL hydrochar obtained from different types of feedstocks (e.g., sludge) is rich in ash (50–70 wt %) and has low C^58,59^ and volatile content.^59^ Substantial differences are observed comparing HC with its corresponding biochar. A decrease in volatiles of approximately 5% is observed between HC and B–HC and results in a comparable increase in ash content, whereas the fixed carbon content remains substantially constant. This is in agreement with the fact that hydrochar results from a previous thermochemical decomposition during bio-crude production.^60–62^ In contrast, the reduction in volatile matter in S is much more pronounced, decreasing from about 70% to 20% from S to B-S due to the contribution of the decomposition of hemicellulose and cellulose fractions. The decomposition of the organic components in S corresponds to a great release of oxygenated compounds in the gas and liquid phases.

The results of the elemental analysis show that HC retains a relatively high oxygen content during HTL, thus resulting in a moderate aromaticity (H/C = 0.88), which is consistent with the literature. In fact, previous studies show the presence of residual oxygenated surface functional groups on the hydrochar surface whose abundance decreases with increasing HTL temperature.^63,64^ Lignin-derived hydrochar was found to be rich in C=C bands with increasing HTL temperature and time,^65^ and Raman analysis of corn stover-derived hydrochar showed an aromatic carbon structure still disordered after thermal treatment.^66^ The relatively higher H/C ratio of B–HC (0.69) compared to the other biochars suggests a lower degree of carbon condensation after pyrolysis at 450 °C. This interpretation is supported by FTIR analysis (Figure 6), which revealed the persistence of residual O–H stretching bands (∼3400 cm^–1^), weak aliphatic C–H contributions (2920–2850 cm^–1^), and C–O vibrations in the 1200–1000 cm^–1^ region. It is likely that the presence of residual acidic functional groups in hydrochar limits the ash alkalizing effect, thus determining a slightly alkaline pH (8.5) of HC despite its high content of AAEMs. The partial carbonization results also in a limited specific surface area (BET = 21.8 m^2^ g^–1^). In contrast, B–HC shows a substantial increase in the surface area (BET = 64.6 m^2^ g^–1^) and a decrease in H/C and O/C ratios, indicating enhanced carbonization and the development of a more condensed aromatic structure during pyrolysis. The higher BET surface area observed for the B–HC can be explained by the reaction environment of HTL, where feedstock is processed in liquid water at high temperature and pressure (typically 250–350 °C), enabling water to act as a reactive solvent that promotes hydrolysis of the organic fraction digestate undergoing dehydration, decarboxylation, and partial aromatization forming the HC amorphous carbon matrix, already containing micro- and mesopores.^67,68^ After pyrolysis of HC, the devolatilization of the residual organic fraction leads to the opening of pre-existing micropores, while the already carbonized structure undergoes less collapse, resulting in an overall increase in the specific surface area. In addition, although catalytic gasification reactions (e.g., C + CO_2_ → 2CO and C + H_2_O → CO + H_2_) are more pronounced at higher temperatures with respect to 450 °C, the presence of ash retained in HC can promote structural evolution and micropore formation in the carbon matrix during subsequent thermal treatment.^69,70^

**Figure 6:**
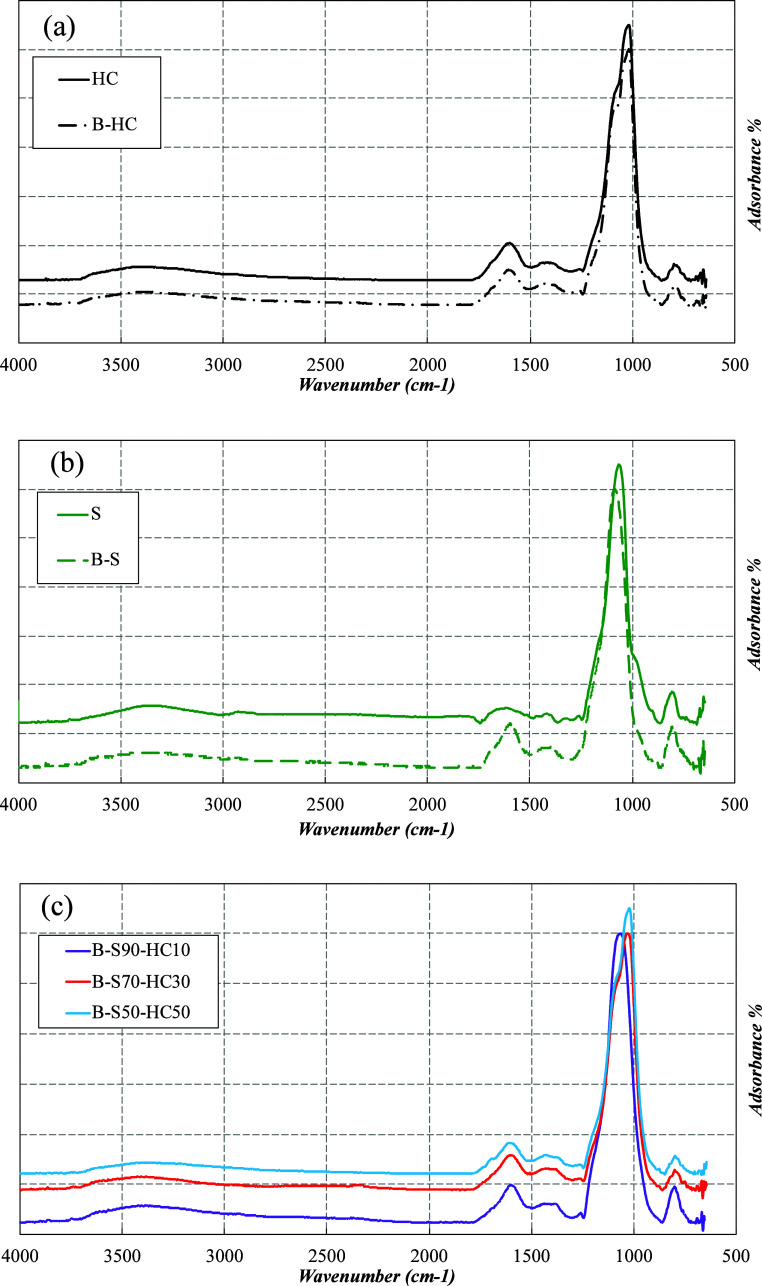
FTIR spectra of feedstocks H (hydrochar), S (straw), and corresponding biochars and blends produced at *T* = 450 °C.

S is characterized by a different pyrolytic behavior. Different from B-HC, B-S is characterized by a substantial reduction of the O/C from 0.73 in S to 0.11 in B-S and a degree of carbonization higher than B-HC, evidenced by the lower H/C value. Compared to B-HC, B-S shows a significantly higher pH (10.9) but lower surface area (BET = 7.7 m^2^ g^–1^). The higher pH can be attributed primarily to the higher concentrations of AAEMs (Table 5), which strongly influence the alkalinity of the biochar^71,72^ due to the formation of carbonates, oxides, and other soluble alkaline mineral phases during pyrolysis, differently from B-HC, where most of the AAEM-based soluble compounds have already been released during the HTL pretreatment. The mineral phases also strongly affect biochar porosity, promoting partial pore blockage or structural collapse, which can explain the limited BET surface area of B-S.^72^ In contrast to B-HC, B-S is produced directly via pyrolysis without any previous hydrothermal pretreatment, and the biomass structure is thermally decomposed at 450 °C, preserving the original structure of plant fibers.

**Table 5:** Ash Composition of HC, S, and all Biochar Produced from Pyrolysis Tests at 450 °C

Inorganic elements content [mg/kg, db]							
	HC	S	B–HC	B–S	B–S90–HC10	B–S70–HC30	B–S50–HC50
Na	2695 ± 404	1001 ± 150	1930 ± 290	2396 ± 359	1916 ± 319	2191 ± 329	2269 ± 340
Mg	14,253 ± 2138	522 ± 78	11,836 ± 1775	1109 ± 166	2497 ± 473	5101 ± 765	6518 ± 978
Al	17,534 ± 2630	448 ± 67	15,901 ± 2385	812 ± 122	4590 ± 618	8210 ± 1232	12,606 ± 1891
P	16,262 ± 2439	1525 ± 229	17,934 ± 2690	3091 ± 464	5407 ± 832	8574 ± 1286	9176 ± 1377
K	12,225 ± 1834	10,025 ± 1504	10,697 ± 1605	34,986 ± 5248	32,018 ± 4215	25,306 ± 3796	21,892 ± 3284
Ca	47,730 ± 7159	972 ± 146	60,707 ± 9106	5230 ± 785	10,792 ± 2322	25,200 ± 3780	32,734 ± 4910
Cr	33 ± 5	1.1 ± 0.1	38 ± 5	3.5 ± 0.5	8 ± 1.2	14 ± 2	22 ± 3
Mn	502 ± 75	32 ± 5	554 ± 83	71 ± 11	147 ± 25	280 ± 42	349 ± 52
Fe	10,632 ± 1595	245 ± 37	10,994 ± 1649	468 ± 70	1873 ± 389	5470 ± 821	7314 ± 1097
Ni	38 ± 6	0.7 ± 0.1	41 ± 6	1.3 ± 0.2	8.3 ± 1.5	22 ± 3.3	34 ± 5
Cu	62 ± 9	15 ± 2	60 ± 9	6 ± 0.9	15 ± 2	29 ± 4.4	45 ± 6.8
Zn	296 ± 45	45 ± 7	375 ± 56	98 ± 14.8	173 ± 22.8	211 ± 31.7	246 ± 36.9
Pb	22 ± 3	0.7 ± 0.1	3.6 ± 0.5	n.d.	13 ± 1.9	14 ± 2	19 ± 2.8
Cd	5 ± 0.75	0.6 ± 0.09	1.3 ± 0.2	0.6 ± 0.09	0.4 ± 0.06	0.3 ± 0.05	0.5 ± 0.08

Blending HC with S at ratios significantly modulates the resulting biochar properties and reflects the combined influence of the organic matrix and the inorganic fraction. Increasing the HC in the blends increases the ash content from 33.1 to 49.6 wt % db in biochars and reduces fixed carbon content from 43.6 to 30.73 wt % db, while a slight effect is observed for the volatiles. Conversely, increasing the HC content in the blends results in only small variations in both the H/C and O/C ratios, consistent with the limited contribution of HC to the final organic fraction of the biochars in the blends with respect to B-S. As a result, the H/C values remain within the range associated with high aromaticity and long-term stability in soil, even in the most extreme case represented by a blend containing 50 wt % HC. Increasing the HC fraction greatly decreases the pH from 10.7 to 9.1, indicating a dilution of the alkaline ash, K and Na, present in straw in higher concentration.^56,57^ Simultaneously, the presence of HC promotes a progressive increase in the surface area compared to B-S from 10.4 to 30.2 m^2^ g^–1^ as HC content increases from 10 wt % to 50 wt %.

FTIR analysis is carried out to investigate the structural changes occurring during pyrolysis of HC, S, and their corresponding biochars and biochar blends obtained at 450 °C. The spectra (Figure 6) of the raw materials showed the typical features of oxygen-rich carbonaceous structures. In particular, HC and S exhibited a broad absorption band around 3400 cm^–1^ attributed to O–H stretching vibrations of hydroxyl groups and adsorbed water. Weak bands in the 2920–2850 cm^–1^ region were assigned to aliphatic C–H stretching vibrations, while the intense bands between 1200 and 1000 cm^–1^ were associated with C–O and C–O–C vibrations from oxygenated functionalities and polysaccharide-derived structures. In the case of straw, the strong contribution around 1030 cm^–1^ is also related to Si–O vibrations associated with the silica-rich ash fraction typical of herbaceous biomass. After pyrolysis, all biochars showed a significant decrease in the intensity of O–H, aliphatic C–H, and C–O bands, indicating dehydration, devolatilization, decarboxylation, and progressive removal of oxygen-containing functional groups during thermal treatment. Simultaneously, the relative contribution of the band around 1580–1600 cm^–1^ became more pronounced, suggesting increased aromatic condensation and carbonization of the solid matrix.

Compared to B-S, the B-HC still exhibited residual O–H and C–O contributions together with weak aliphatic C–H bands, indicating the persistence of part of the oxygen-containing surface functionalities at 450 °C. This observation is consistent with the elemental analysis results, since B-HC showed a relatively higher H/C ratio (0.69) than B-S (0.54), suggesting a lower degree of carbon condensation. The biochars produced from mixtures exhibited intermediate spectral features between B-S and B-HC. Increasing the HC ratio in the blends progressively reduced the intensity of oxygenated bands while maintaining the aromatic contribution around 1600 cm^–1^, suggesting that the presence of hydrochar influenced the structural evolution of the carbon matrix during copyrolysis. Overall, the FTIR results support the trends observed from elemental analysis and confirm the reduction of oxygen-containing functionalities due to the thermal treatment.

SEM images, reported in Figure 7, reveal a clear morphological evolution of S and HC after the pyrolysis treatment, showing the progressive degradation of the lignocellulosic structure, fragmentation of fibers, and accumulation of mineral-rich surface deposits. S (Figure 7, panel a) exhibits a compact fibrous morphology with well-preserved longitudinal bundles, while B-S (Figure 7, panel c) shows increased surface roughness, fractures, and partial structural collapse due to devolatilization. However, no significant microporosity development is observed, likely because of the relatively low pyrolysis temperature and the accumulation of mineral-rich surface deposits typical of straw. HC (Figure 7, panel b) displays a markedly different morphology characterized by irregular sponge-like aggregates and highly heterogeneous structures associated with hydrothermal carbonization. After pyrolysis, B-HC (Figure 7, panel d) shows highly fragmented and mineral-rich structures. Blended S/HC-derived biochars exhibit intermediate behaviors as shown for B-S50-HC50 where both the S-derived and HC-derived structures are visible (Figure 7, panels e and f), respectively. However, no interactions were observed between the two structures.

**Figure 7:**
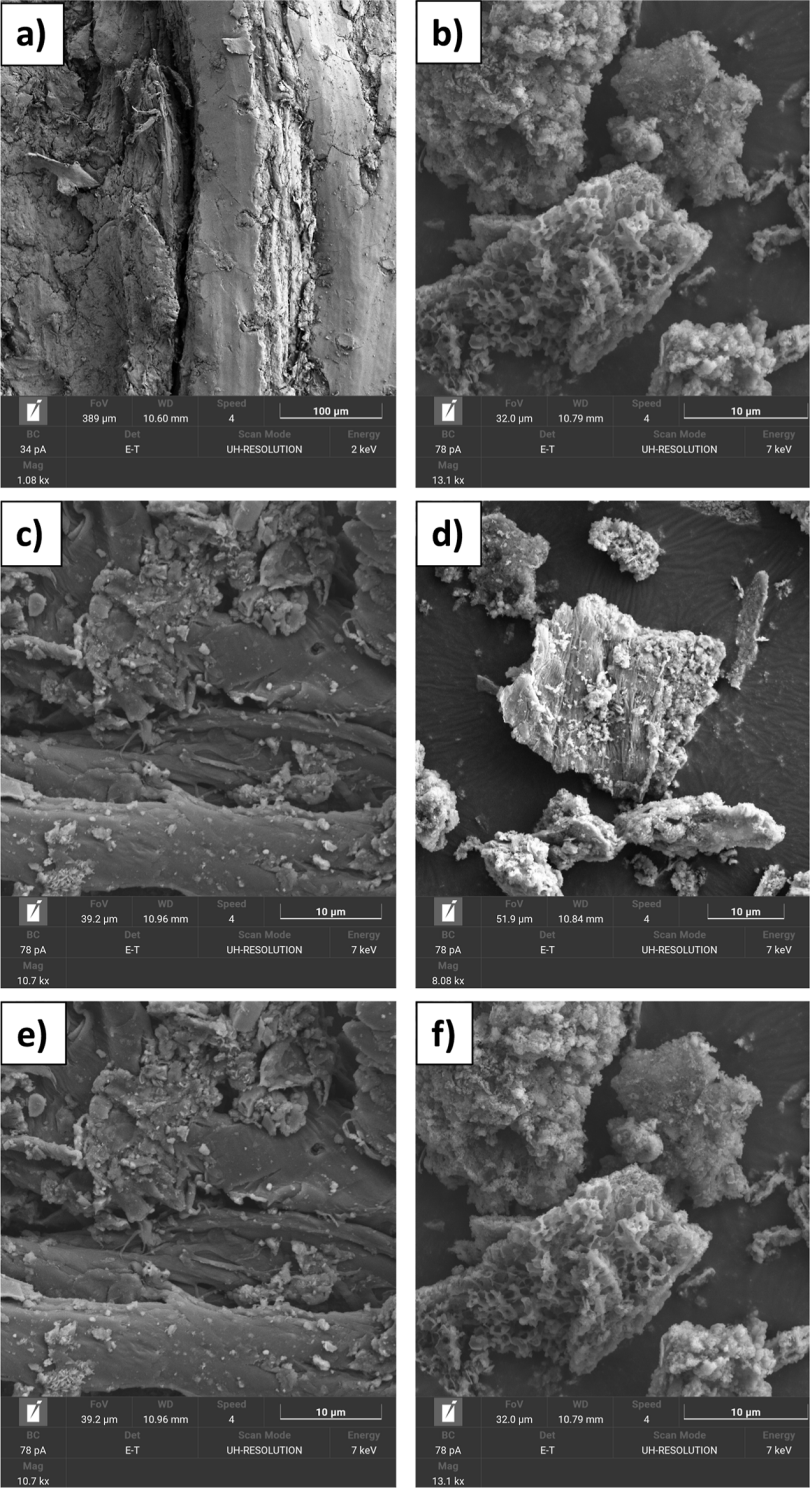
Scanning electron micrographs of (a) S, (b) HC, (c) B-S, (d) B-HC, and (e,f) B-S50-HC50 are shown at different magnifications.

The ash compositions of HC from HTL and all biochars are reported in Table 5. The inorganic elements are particularly relevant because they can act as potential sources of plant nutrients when biochars are applied to soil contributing to soil fertility and nutrient cycling.^73^ There are great differences in most of the metal contents in the raw materials HC and S. Different from B-S, where the inorganics present in the feedstock concentrate during pyrolysis, most of the inorganics in HC remain stable or decrease in concentration after the thermal treatment. This behavior is probably due to the different chemical forms of the inorganic in the digestate as well as in HC due to the hydrothermal preliminary treatment. During HTL, a large fraction of K is transferred to the aqueous phase due to its high solubility, as a consequence, the K chemical species retained in HC may not only include mineralized or precipitated forms trapped within the mineral matrix but also less stable forms that can be released under certain conditions.^60,74^

The biochars obtained from blends of HC and S at different ratios displayed intermediate inorganic compositions, reflecting the different nature of the original feedstocks and conversion pathways, suggesting that coprocessing can modulate the ash chemistry, thus affecting the fertilizing ability of biochars.

Concerning potentially toxic elements, those reported in the EU regulation (Pb, Cu, Zn, and Cd) are detected in the biochars from HC and S in concentrations lower than the required limits, namely, Pb < 120 mg/kg, Cu < 230 mg/kg, Zn < 500 mg/kg, and 1.5 mg/kg for Cd.^75^ The concentration in the biochars derived from HC-S blends generally increases as the HC concentration increases in the blend. However, all the biochars meet the regulatory limits even in the extreme case represented by a blend containing 50 wt % HC.

### 3.4 Pyrolysis Gas

To elucidate the effect of increasing HC, and thus inorganic content, during pyrolysis, on the gas production and composition, the properties of the gas phase and the release gas curves during the process as a function of the pyrolysis temperature are reported in Table 6 and Figure 8, respectively.

**Table 6:** Yields, Composition, and HHV of the Pyrolysis Gaseous Product

	CH_4_	CO	CO_2_	H_2_	CH_4_	CO	CO_2_	H_2_	HHV
	Gas yield wt %				Gas composition wt %				MJ/kg
HC	0.2	0.7	4.2	0.01	4.2	13	81.5	0.3	4.1
S	0.4	4.4	10.2	0.01	2.3	29.2	67.3	0.1	4.3
S90–HC10	0.3	4.3	13	0.01	2.10	26.0	70.7	0.07	3.9
S70–HC30	0.3	3.5	8.7	0.01	2.62	27.2	68.8	0.09	4.3
S50–HC50	0.2	2.7	7.1	0.01	2.3	26.6	69.7	0.1	3.7

**Figure 8:**
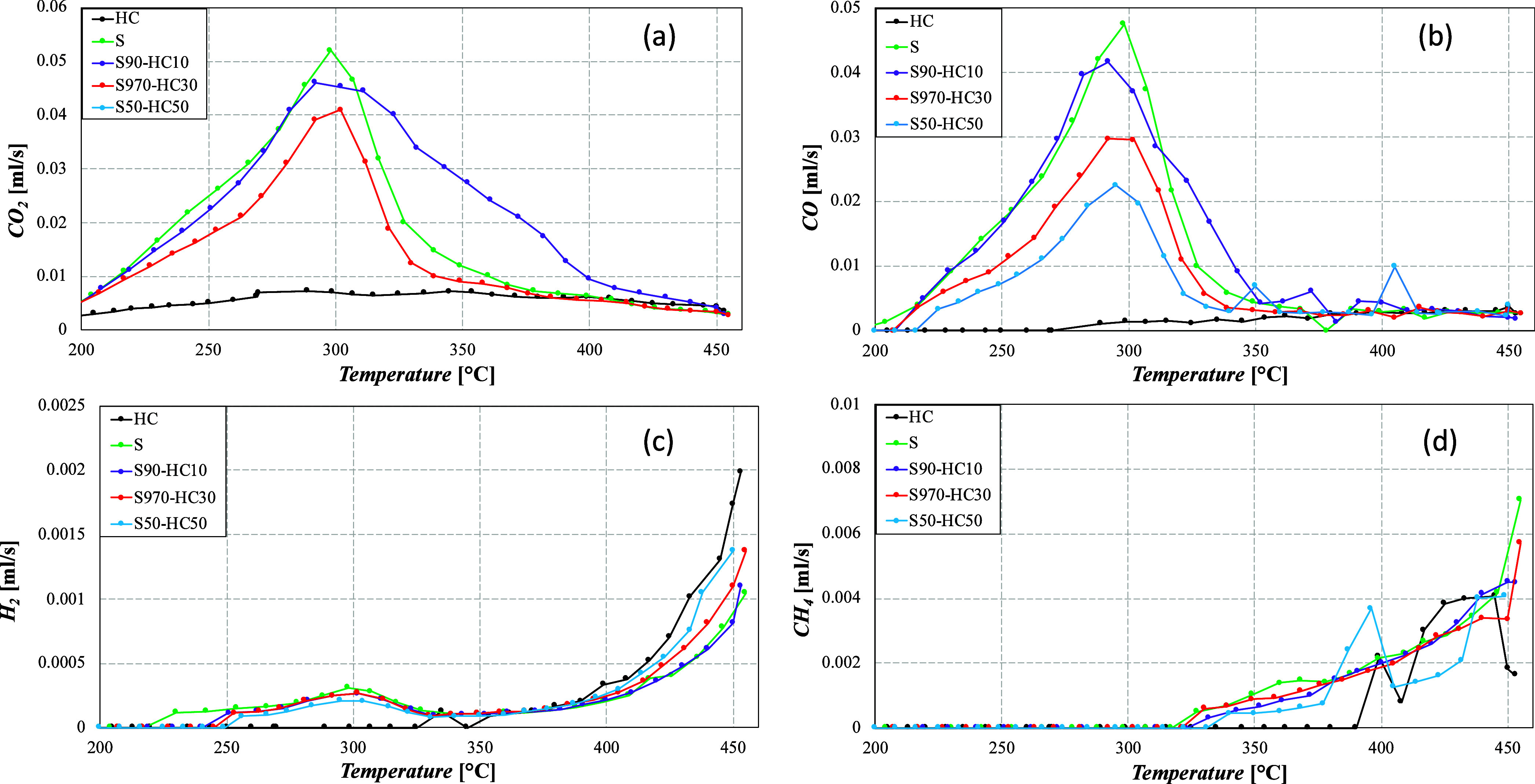
Release rate of the (a) CO_2_, (b) CO, (c) H_2_, and (d) CH_4_ as a function of temperature (mL s^–1^ g^–1^ biomass on daf basis at STP conditions).

In detail, as shown in Table 6, CO_2_ is the dominant gaseous species for all samples, ranging from 81.5 to 67.3 wt %, followed by CO varying from 29.2 to 13.0 wt %. CH_4_ is present in lower amounts, and H_2_ remains lower than 0.3 wt %.

The higher CO in S reflects the decomposition of hemicellulose and cellulose and the cleavage of carbonyl and ether groups. In contrast, a markedly higher CO_2_ concentration in the case of HC is observed in agreement with previous findings showing that pyrolysis gas from different types of hydrochars is mainly composed of CO_2_.^58,76,77^ CO_2_ release can be attributed to decarboxylation of oxygenated functionalities enriched during digestate HTL conversion into HC^78^ and to the catalytic role of the great amount of AAEMs in promoting char–CO_2_ forming reactions.^46^ Among the limited studies on the thermal treatment of HTL-derived hydrochar, Na and K were identified as the primary catalysts for CO_2_ formation.^58^

Increasing the HC content in the blends generally shifts the composition toward higher CO_2_ and slightly lower CO and light hydrocarbon contents. However, given the low contribution of HC to gas formation, the composition of the gas during the copyrolysis of S and HC remains substantially stable across the different S to HC ratios in the blends.

This compositional shift is reflected in the higher heating value of the gaseous mixture, with blends showing intermediate or slightly lower values, consistent with the dilution of combustible species (CO, CH_4_, H_2_) by noncombustible CO_2_.

The gas release curves further highlight different behaviors of S, HC, and the blends. For S and blends, CO_2_ and CO evolution exhibit pronounced peaks in the range of 290–310 °C. For S, the peak is at 300 °C, corresponding to the active devolatilization region of hemicellulose and cellulose.^79^

In contrast, HC shows a much flatter and lower-intensity release profile, reflecting its more aromatized and thermally stable structure. The presence of inorganic elements, K and Ca, significantly affects these devolatilization pathways.^46,80^ The pyrolysis release curves of S and HC blends show that the presence of HC in low amounts (S90–HC10) promotes reactions leading to the formation of CO_2_, especially at *T* > 300 °C. This is likely due to the increased content of AAEMs facilitating secondary reactions that promote the overall gas production during pyrolysis as already observed in the literature for K-rich biomass compared to the corresponding demineralized form.^81^

However, as the percentage of S decreases and the fraction of HC increases, this promoting effect becomes progressively less pronounced. In blends with a lower S content, the overall gas production tends to be more limited because HC inherently produces relatively small amounts of gaseous species during pyrolysis. Consequently, the increasing presence of HC reduces the overall intensity of gas-releasing reactions, masking the catalytic or promoting role exerted by S that is clearly observable at higher S concentrations.

For both S and HC, CH_4_ formation starts at higher temperatures, 330 and 390 °C for S and HC, respectively. CH_4_ release is typically attributed to the cracking of lignin-derived fragments. It is likely that in HC, the more reactive functionalities of the lignin component have already been decomposed during the HTL, and higher temperatures are required to further decompose more thermally stable fragments. Moreover, the release of CH_4_ in the case of HC occurs in two stages, showing two different peaks at 400 and 435 °C, whereas the release rate curve in the case of S increases monotonically. The formation of CH_4_ at low temperature was already observed in the literature for the pyrolysis of sludge-derived hydrochars and was mainly attributed to the cleavage of aliphatic side chains.^82^ As the pyrolysis temperature increases, the vapors undergo secondary cracking reactions at higher temperatures, leading to the further generation of CH_4_. This behavior is reflected in the blends only in the sample S50-HC50, whereas it disappears for lower concentrations of HC in the starting material due to the low contribution of HC to gas formation. H_2_ release is observed, even though in limited amounts, even at temperatures as low as 200 °C in the case of S and at 330 °C in the case of HC, whereas the onset of H_2_ release is observed at intermediate temperatures in the case of blends, thus revealing a relevant effect of the presence of HC even in low concentration. In all cases, H_2_ increases sharply above 400 °C, especially for HC, likely due to secondary char reactions and aromatization processes, which are catalyzed by the high content of AAEMs.^46^

## 4 Conclusions

In this work, the HTL process of digestate was investigated for bio-crude production, and pyrolysis was studied as a pathway to valorize the solid residue from HTL, namely, hydrochar. HTL process conditions (temperature and process time) were optimized based on bio-crude yields and composition. Furthermore, in the pyrolysis process, the effect of blends of hydrochar and straw at different concentrations was evaluated on biochar properties. Overall, the results showed that:

- The best operating conditions investigated in this study (350 °C and 10 min) allowed to obtain a bio-crude with a H/C ratio of 1.3 and an O/C ratio of 0.3. In addition, a bio-crude yield (on a dry and ash-free basis) of about 30%, with an associated energy recovery of 50% (HHV of 28 MJ/kg), was found.
- GC–MS analyses showed that all bio-crude obtained under different operating conditions are mainly characterized by the presence of phenolic, O- and N-heterocyclic compounds, and hydrocarbons, making the bio-crude particularly attractive for SAF production, if suitably upgraded.
- Hydrochar pyrolysis at 450 °C produced mainly biochar due to its high ash content and aromatic structure, resulting in limited liquid and gas formation, whereas straw yielded lower biochar and higher liquid and gas fractions.
- Biochar from hydrochar shows moderate aromaticity, slightly alkaline pH, and moderate BET surface area.
- Copyrolysis of hydrochar and straw showed intermediate product distributions, with biochar yield increasing and liquid yield decreasing as the hydrochar fraction increased. With increasing hydrochar in blends, BET surface and ash content increase, fixed carbon and pH decrease, while volatiles, H/C, and O/C ratios remain almost stable within the range associated with aromatic biochars. Biochars from the blends show intermediate inorganic compositions, with P increasing with hydrochar content and K decreasing accordingly and potentially improved fertilizer properties.
- Some deviations from an additive dependence of product yields on blend composition were observed due to interactions between straw and hydrochar inorganics during copyrolysis.

Overall, all of the biochar formulations meet the limits imposed by EU regulations for biochar as soil amendment.

These results support the energy valorization of digestate by HTL to obtain a liquid biofuel and the valorization of HTL-derived hydrochar via copyrolysis with lignocellulosic biomass in an integrated biorefinery scheme.

## Supplementary Material



## Notes

Identification of the organic compounds obtained from GC–MS analyses carried out on bio-crude samples attained during the HTL test of digestate (PDF)
