# Supplementary material for: Hydrothermal Liquefaction of Digestate and Copyrolysis of Hydrochar–Straw Blends: An Integrated Approach for Bio-crude and Biochar Production
Source: Energy Fuels. 2026 Jul 17;40(30):16479–95. doi: 10.1021/acs.energyfuels.6c01322 (PMC13435599; doi:10.1021/acs.energyfuels.6c01322)
Supplement: Supplementary file 1 [file ef6c01322_si_001.pdf]

Supplementary Materials

Hydrothermal Liquefaction of Digestate and Co-Pyrolysis of Hydrochar–Straw Blends: An Integrated Approach for Bio-crude and Biochar Production

Corinna Maria Grottola[a], Giusy Marotta[a,b], Davide Amato[a], Francesca Di Lauro[c,a], Marco Balsamo[b], Fabio Montagnaro[b,a], Roberto Solimene[a], Paola Giudicianni[a], Raffaele Ragucci[a]*

[a]Istituto di Scienze e Tecnologie per l'Energia e la Mobilità Sostenibili (CNR-STEMS), Naples 80125, Italy

[b] Dipartimento di Scienze Chimiche, Università degli Studi di Napoli Federico II, Naples 80126, Italy

[c]Dipartimento di Ingegneria Chimica, dei Materiali e della Produzione Industriale, Università degli Studi di Napoli Federico II 80126, Naples, Italy

Corresponding Author

*Francesca Di Lauro – [c]Dipartimento di Ingegneria Chimica, dei Materiali e della Produzione Industriale, Università degli Studi di Napoli Federico II, Naples, Italy

Email: francesca.dilauro2@unina.it

Organic compounds determined in the bio-crude.

Table S1. Main compounds identified by the GC−MS analyses carried out on bio-crude samples attained during HTL test of digestate at 350°C and 10 min.

**Aldehydes/Ketones**	**Area %**
2-Butanone	0.44
2-Pentanone	0.18
Cyclopentanone	0.58
Cyclopentanone, 2-methyl-	0.21
2-Cyclopenten-1-one	0.18
(R)-(+)-3-Methylcyclopentanone	0.19
2-Cyclopenten-1-one, 2-methyl-	1.69
3-Ethylcyclopentanone	0.15
2-Cyclopenten-1-one, 3-methyl-	1.61
2-Cyclopenten-1-one, 2,3-dimethyl-	1.11
2-Cyclopenten-1-one, 3,4-dimethyl-	0.43
2-Cyclopenten-1-one, 3,4-dimethyl-	0.56
2-Cyclopenten-1-one, 2,3-dimethyl-	1.69
2-Cyclopenten-1-one, 2,3,4-trimeth 1	0.27
Acetophenone	0.21
Ethanone, 1-(1H-pyrrol-2-yl)-	0.26
Ethanone, 1-(1-cyclohexen-1-yl)-	0.64
Ethanone, 1-(2-thienyl)-	0.71
Cyclopentanecarboxaldehyde, 2-methyl-3-methylene-	0.15
Ethanone, 1-(2-hydroxyphenyl)-	0.40
1H-Inden-1-one, 2,3-dihydro-	0.23
7-Methylindan-1-one	0.23
**Tot.**	**12.08**
**Hydrocarbons**	
Toluene	0.33
Ethylbenzene	0.23
Styrene	0.26
1-Undecene	0.10
Spiro[2.4]heptane, 4-methylene-	0.11
1-Tridecene	0.45
Cycloundecane, (1-methylethyl)-	0.53
Cetene	0.34
4-Ethylbiphenyl	1.04
4-Propyl-1,1'-diphenyl	1.41
cis-2-Methyl-7-octadecene	0.50
9-Tricosene, (Z)-	0.19
**Tot.**	**5.49**
**N-containing heterocycles**	
Pyrazine	0.17

Pyridine	1.47
Pyridine, 2-methyl-	0.39
1H-Pyrrole, 1-ethyl-	0.17
Pyrazine, methyl-	0.90
Pyridine, 3-methyl-	0.46
Pyridine, 4-methyl-	0.15
Pyridine, 2-ethyl-	0.17
Pyrazine, 2,5-dimethyl-	0.67
Pyrazine, ethyl-	0.55
Pyrazine, 2,3-dimethyl-	0.24
Pyridine, 3,5-dimethyl-	0.28
Pyridine, 2,3-dimethyl-	0.17
Pyridine, 3-ethyl-	0.22
Pyrazine, 2-ethyl-6-methyl-	0.17
Pyrazine, 2-ethyl-3-methyl-	0.06
1H-Pyrrole, 2,3,5-trimethyl-	0.12
Pyrazine, 2,6-diethyl-	0.15
2-Pyrrolidinone, 1-methyl-	0.62
Quinoline	0.45
Indole	0.63
Quinoline, 4-methyl-	0.43
1H-Indole, 4-methyl-	0.35
1H-Indole, 5-methyl-	0.13
Pyridine, 4-phenyl-	0.15
1H-Indole, 1,2-dimethyl-	0.60
5,10-Diethoxy-2,3,7,8-tetrahydro-1H,6H-dipyrrolo[1,2-a:1',2'-d]pyrazine	0.83
**Tot.**	**10.68**
**Organic acid**	
Dodecanoic acid	0.23
Tetradecanoic acid	0.40
Pentadecanoic acid	0.36
Tridecanoic acid	0.28
n-Hexadecanoic acid	1.29
9-Octadecenoic acid, (E)-	0.79
Octadecanoic acid	0.37
**Tot.**	**3.72**
**Phenolic compounds**	
Phenol	4.91
Phenol, 2-methoxy-	11.05
Phenol, 2-methyl-	0.60
p-Cresol	2.59
2-Methoxy-6-methylphenol	0.67
Phenol, 2-ethyl-	0.33
Phenol, 2,4-dimethyl-	0.57
Creosol	2.72
Phenol, 4-ethyl-	5.11

Phenol, 4-ethyl-2-methoxy-	4.26
m-Guaiacol	0.27
Phenol, 2-propyl-	1.19
Phenol, 2,6-dimethoxy-	5.19
3-Allyl-6-methoxyphenol	0.18
Apocynin	0.51
Acetophenone, 4'-hydroxy-	0.78
(+-)-2-Phenethanamine, 1-methyl-N-vanillyl-	0.47
**Tot.**	**41.40**
**Others**	
Disulfide, dimethyl	0.36
Benzene, (ethylthio)-	0.11
Benzene, 1-methyl-3,5-bis[(trimethylsilyl)oxy]-	0.26
1,2,3-Trimethoxybenzene	0.71
9-Chloromethyl-S-octahydrophenanthrene	0.18
9-Octadecenamide, (Z)-	0.59
**Tot.**	**2.21**
**Unknown**	**24.42**
